# A new Sumudu transform iterative method for time-fractional Cauchy reaction–diffusion equation

**DOI:** 10.1186/s40064-016-2426-8

**Published:** 2016-06-24

**Authors:** Kangle Wang, Sanyang Liu

**Affiliations:** School of Mathematics and Statistics, Xidian University, Xi’an, 710118 China

**Keywords:** Sumudu transform, Caputo fractional derivative, Diffusion equation

## Abstract

In this paper, a new Sumudu transform iterative method is established and successfully applied to find the approximate analytical solutions for time-fractional Cauchy reaction–diffusion equations. The approach is easy to implement and understand. The numerical results show that the proposed method is very simple and efficient.

## Background

The fractional differential equations have gained a lot of attention of physicists, mathematicians and engineers in the past two decades (Oldham and Spanier 1974; Hilfer 2000; Kilbas et al. 1993; Podlubny 1999; Debnath 1997; Yang and Srivastava 2015; Yang et al. 2015b, c, d; Wang et al. 2014, 2015a, b, c; Jiwari and Mittal 2011). All kinds of interdisciplinary problems can be modeled with the help of fractional derivatives. However, it is very difficult for us to find their exact solutions to most fractional differential equations, so numerical and approximation methods have to be used. So far, many methods have been used to solve linear and nonlinear fractional differential equations. For example, the Adomain decomposition method (ADM) (Wazwaz 1999), the homotopy perturbation method (HPM) (He 1999), the variational iteration method (VIM) (Safari et al. 2009), homotopy analysis method (HAM) (Liao 1992, 2004) and differential quadrature method (Jiwari et al. 2012). The time-fractional Cauchy reaction–diffusion equation (Verma et al. 2014; Jiwari et al. 2014; Mittal and Jiwari 2011) is one of all the important fractional partial differential equations. The time-fractional Cauchy reaction–diffusion equations can be used to describe many kinds of linear and nonlinear systems in chemistry, physics, ecology, biology and engineering (Britton 1998; Grindrod 1996). Kumar (2013) have obtained the approximate solutions of time-fractional Cauchy reaction–diffusion equations by using the homotopy perturbation transform method with the help of Laplace transform. In Gejji and Jafari (2006), proposed NIM for solving linear and nonlinear integral and differential equation. The NIM is very easy to understand and implement and obtain better result than existing methods.

In this paper, we establish a new Sumudu transform iterative method (NSTIM) with the help of the Sumudu transform (Chaurasia and Singh 2010) for obtaining analytical and numerical solutions of the time-fractional Cauchy reaction–diffusion equations. Our iterative method is new and generalizes NIM due to Gejji and Jafari (2006). The advantage of this new method which we proposed is to make the calculation simple and highly accurate to approximate the exact solution.

## Basic definition

In this section, we give some basic definitions and properties of fractional calculus and Sumudu transform, which we will use in this paper:

### **Definition 1**

A real function $f(x),\,x>0$, is said to be in the space $C_{\mu }$, $\mu \in {R}$ if there exists a real number $p,\,(p>\mu )$, such that $f(x)=x^{p}f_{1}(x)$, where $f_{1}(x)\in {C[0,\infty )}$, and it is said to be in the space $C_{\mu }^{m}$ if $f^{(m)}\in {C_{\mu }}$, $m\in {N}$ (Dimovski 1982).

### **Definition 2**

The Riemann–Liouville fractional integral operator of order $\alpha \ge 0$, of a function $f(x)\in {C_{\mu }}$, $\mu \ge -1$ is defined as (Hilfer 2000; Yang et al. 2015a)

1 $$\begin{aligned} I^{\alpha }f(x)= \left\{ \begin{array}{ll} \frac{1}{\Gamma (\alpha )}\int _{0}^{x}(x-t)^{\alpha -1}f(t)dt,&{} \quad \alpha>0,\,\,x>0,\\ I^{0}f(x)=f(x),&{}\quad \alpha =0, \end{array} \right. \end{aligned}$$

where $\Gamma (\cdot )$ is the well-known Gamma function.

Properties of the operator $I^{\alpha }$, which we will use here, are as follows

For $$f\in {C_{\mu }},\,\mu ,\gamma \ge -1,\,\alpha ,\beta \ge 0$$,

$$\begin{aligned}&(1)\,I^{\alpha }I^{\beta }=I^{\beta }I^{\alpha }f(x)=I^{\alpha +\beta }f(x),\\&(2)\,I^{\alpha }x^{\gamma }=\frac{\Gamma (\gamma +1)}{\Gamma (\alpha +\gamma +1)}x^{\alpha +\gamma }. \end{aligned}$$

### **Definition 3**

The fractional derivative of *f*(*x*) in the Caputo sense is defined as (Hilfer 2000; Yang et al. 2015a)

2 $$\begin{aligned} D^{\alpha }f(x)=I^{n-\alpha }D^{n}f(x)=\frac{1}{\Gamma (n-\alpha )}\int _{0}^{x}(x-t)^{n-\alpha -1}f^{(n)}(t)dt, \end{aligned}$$

where $$n-1<\alpha \le {n},\,n\in {N},\,x>0,\,f\in {C_{-1}^{n}}$$.

The following are the basic properties of the operator $D^{\alpha }$:

$$\begin{aligned}&(1)\,D^{\alpha }I^{\alpha }f(x)=f(x),\\&(2)\,I^{\alpha }D^{\alpha }f(x)=f(x)-\sum _{k=0}^{n-1}f^{(k)}(0^{+})\frac{x}{k!},\quad x>0. \end{aligned}$$

### **Definition 4**

The Mittag–Leffler function $E_{\alpha }$ with $\alpha >0$ is defined as (Chaurasia and Singh 2010)

3 $$\begin{aligned} E_{\alpha }(z)=\sum _{n=0}^{\infty }\frac{z^{\alpha }}{\Gamma (n\alpha +1)}. \end{aligned}$$

### **Definition 5**

The Sumudu transform is defined over the set of function $A=\{f(t)|\exists {M},\tau _{1},\tau _{2}>0,|f(t)|<Me^{\frac{|t|}{\tau _{j}}}, if t\in (-1)^{j}\times [0,\infty )\}$ by the following formula (Chaurasia and Singh 2010)

4 $$\begin{aligned} S[f(t)]=\int _{0}^{\infty }e^{-t}f(vt)dt,\quad v\in (-\tau _{1},\tau _{2}). \end{aligned}$$

### **Definition 6**

The Sumudu transform of the Caputo fractional derivative is defined as (Chaurasia and Singh 2010)

5 $$\begin{aligned} S[D_{x}^{n\alpha }u(x,t)]=v^{-n\alpha }S[u(x,t)]-\sum _{k=0}^{n-1}v^{(-n\alpha +k)}u^{(k)}(0,t),\quad n-1<n\alpha \le {n}. \end{aligned}$$

## The new Sumudu transform iterative method (NSTIM)

To illustrate the basic idea of the NSTIM for the fractional partial differential equation, we consider the following equation with the initial condition as

6 $$\left\{ \begin{array}{lll} &D_{t}^{n\alpha }u(x,t)+Lu(x,t)+Ru(x,t)=g(x,t),\\ &n-1<n\alpha \le {n},\\ &u(x,0)=h(x). \end{array} \right.$$

where $D_{t}^{n\alpha }$ is the Caputo fractional derivative operator, $D_{t}^{n\alpha }=\frac{\partial ^{n\alpha }}{\partial {t}^{n\alpha }}$, *L* is a linear operator, *R* is general nonlinear operator, *g*(*x*, *t*) is continuous function.

Applying Sumudu transform on both sides of Eq. (6), we get

7 $$\begin{aligned} S\left[ D_{t}^{n\alpha }u(x,t)\right] +S\left[ Lu(x,t)+Ru(x,t)\right] =S[g(x,t)]. \end{aligned}$$

Using the property of the Sumudu transform, we can obtain

8 $$\begin{aligned} S[u(x,t)]-v^{n\alpha }\sum _{k=0}^{n-1}u^{(k)}(x,0)+v^{n\alpha }S[Lu(x,t)+Ru(x,t)-g(x,t)]=0. \end{aligned}$$

On simplifying (8), we have

9 $$\begin{aligned} S[u(x,t)]=v^{n\alpha }\sum _{k=0}^{n-1}u^{(k)}(x,0)-v^{n\alpha }S[Lu(x,t)+Ru(x,t)-g(x,t)]. \end{aligned}$$

Operating the inverse Sumudu transform on both sides of Eq. (9), we get

10 $$\begin{aligned} u(x,t)=S^{-1}\left[ v^{n\alpha }\sum _{k=0}^{n-1}u^{(k)}(x,0)\right] -S^{-1}[v^{n\alpha }S[Lu(x,t)+Ru(x,t)-g(x,t)]]. \end{aligned}$$

Next assume that

$$\left\{ \begin{array}{lll} &{}f(x,t)=S^{-1}[v^{n\alpha }\sum _{k=0}^{n-1}u^{(k)}(x,0)+v^{\alpha }S[g(x,t)]],\\ &{}N(u(x,t))=-S^{-1}[v^{n\alpha }S[Ru(x,t)]],\\ &{}K(u(x,t))=-S^{-1}[v^{n\alpha }S[Lu(x,t)]]. \end{array} \right.$$

Thus, Eq. (10) can be written in the following form

11 $$\begin{aligned} u(x,t)=f(x,t)+K(u(x,t))+N(u(x,t)), \end{aligned}$$

where *f* is a known function, *K* and *N* are given linear and nonlinear operator of *u*, respectively. The solution of Eq. (11) can be written in the series form

12 $$\begin{aligned} u(x,t)=\sum _{i=0}^{\infty }u_{i}(x,t), \end{aligned}$$

we have

13 $$\begin{aligned} K\left( \sum _{i=0}^{\infty }u_{i}\right) =\sum _{i=0}^{\infty }K(u_{i}). \end{aligned}$$

The nonlinear operator *N* is decomposed as (see Gejji and Jafari 2006)

14 $$\begin{aligned} N\left( \sum _{i=0}^{\infty }u_{i}\right) =N(u_{0})+\sum _{i=0}^{\infty }\left\{ N\left( \sum _{j=0}^{i}u_{j}\right) -N\left( \sum _{j=0}^{i-1}u_{j}\right) \right\} . \end{aligned}$$

Therefore, Eq. (11) can be represented as the following form

15 $$\begin{aligned} \sum _{i=1}^{\infty }u_{i}=f+\sum _{i=0}^{\infty }K(u_{i})+N(u_{0}) +\sum _{i=0}^{\infty }\left\{ N\left( \sum _{j=0}^{i}u_{j}\right) -N\left( \sum _{j=0}^{i-1}u_{j}\right) \right\} . \end{aligned}$$

Defining the recurrence relation

16 $$\left\{ \begin{array}{lll} &{}u_{0}=f,\\ &{}u_{1}=K(u_{0})+N(u_{0}),\\ &{}u_{m+1}=K(u_{m})+N\left( u_{0}+\cdots +u_{m}\right) -N\left( u_{0}+u_{1}+\cdots +u_{m-1}\right) , \end{array} \right.$$

we have

17 $$\begin{aligned} (u_{1}+\cdots +u_{m+1})=K(u_{0}+\cdots +u_{m})+N(u_{0}+\cdots +u_{m}). \end{aligned}$$

Namely

18 $$\begin{aligned} \sum _{i=0}^{\infty }u_{i}=f+K\left( \sum _{i=0}^{\infty }u_{i}\right) +N\left( \sum _{i=0}^{\infty }u_{i}\right) . \end{aligned}$$

The *m*-term approximate solution of Eq. (11) is given by

19 $$\begin{aligned} u=u_{0}+u_{1}+u_{2}+u_{3}+\cdots +u_{m-1}. \end{aligned}$$

Similarly, the convergence of the NSTIM, we can refer the paper Gejji and Jafari (2006 and Bhalekar and Gejji (2011).

## Numerical examples

### *Example 1*

Consider the following time-fractional Cauchy reaction–diffusion equation (Kumar 2013)

20 $$\left\{ \begin{array}{lll} &{}u_{t}^{\alpha }(x,t)=u_{xx}(x,t)-u(x,t),\\ &{}0<\alpha \le 1,\\ &{}u(x,0)=e^{-x}+x. \end{array} \right.$$

Applying Sumudu transform on both sides of Eq. (20) and using the differential property of Sumudu transform, we obtain

21 $$\begin{aligned} S[u]=u(x,0)+v^{\alpha }S[u_{xx}-u]. \end{aligned}$$

Using the inverse Sumudu transform on both sides of Eq. (21), we get

22 $$\begin{aligned} u(x,t)=S^{-1}[(e^{-x}+x)]+S^{-1}[v^{\alpha }S[u_{xx}-u]], \end{aligned}$$

namely

23 $$\begin{aligned} u(x,t)=e^{-x}+x+S^{-1}[v^{\alpha }S[u_{xx}-u]]. \end{aligned}$$

According to the NSTIM, we have

$$\left\{ \begin{array}{ll} &{}u_{0}=e^{-x}+x,\\ &{}K[u(x,t)]=S^{-1}[v^{\alpha }S[u_{xx}-u]]. \end{array} \right.$$

By iteration, the following results are obtained

$$\begin{aligned}&u_{0}=e^{-x}+x,\\&u_{1}=-\frac{xt^{\alpha }}{\Gamma (\alpha +1)},\\&u_{2}=\frac{xt^{2\alpha }}{\Gamma (2\alpha +1)},\\&u_{3}=-\frac{xt^{3\alpha }}{\Gamma (3\alpha +1)},\\&u_{4}=\frac{xt^{4\alpha }}{\Gamma (4\alpha +1)},\\&\ldots \ldots \\&u_{n}=(-1)^{n}\frac{xt^{n\alpha }}{\Gamma (n\alpha +1)}. \end{aligned}$$

Therefore, we have the solution of the problem as follows

24 $$\begin{aligned} u(x,t)&=e^{-x}+x\left( 1-\frac{t^{\alpha }}{\Gamma (\alpha +1)}+\frac{t^{2\alpha }}{\Gamma (2\alpha +1)}-\frac{t^{3\alpha }}{\Gamma (3\alpha +1)} +\cdots +(-1)^{n}\frac{t^{n\alpha }}{\Gamma (n\alpha +1)}\right) \nonumber \\&=e^{-x}+xE_{\alpha }(-t^{\alpha }). \end{aligned}$$

The Eq. (24) is approximate to the form $u(x,t)=e^{-x}+xe^{-t}$ for $\alpha =1$, which is the exact solution of Eq. (20) for $\alpha =1$. The result is same as HPTM (Kumar 2013).

### *Example 2*

We consider the following time-fractional Cauchy reaction–diffusion equation (Kumar 2013) as follows

25 $$\left\{ \begin{array}{lll} &{}u_{t}^{\alpha }(x,t)=u_{xx}(x,t)-(1+4x^{2})u(x,t),\\ &{}0<\alpha \le 1,\\ &{}u(x,0)=e^{x^{2}}. \end{array} \right.$$

Applying Sumudu transform on both sides of Eq. (25) and using the differential property of Sumudu transform, we obtain

26 $$\begin{aligned} S[u]=u(x,0)+v^{\alpha }S\left[ (u_{xx}(x,t)-(1+4x^{2})u(x,t))\right] . \end{aligned}$$

Using the inverse Sumudu transform on both sides of Eq. (26), we have

27 $$\begin{aligned} u(x,t)=e^{x^{2}}+S^{-1}[v^{\alpha }\left[ S\left[ (u_{xx}(x,t)-(1+4x^{2})u(x,t))\right] \right] . \end{aligned}$$

According to the NSTIM, we can obtain

$$\left\{ \begin{array}{ll} &{}u_{0}=e^{x^{2}},\\ &{}K[u(x,t)]=S^{-1}[v^{\alpha }\left[ S[(u_{xx}(x,t)-(1+4x^{2})u(x,t))]\right] . \end{array} \right.$$

By iteration, we get the following results as

$$\begin{aligned}&u_{0}=e^{x^{2}},\\&u_{1}=\frac{e^{x^{2}}t^{\alpha }}{\Gamma (\alpha +1)},\\&u_{2}=\frac{e^{x^{2}}t^{2\alpha }}{\Gamma (2\alpha +1)},\\&u_{3}=\frac{e^{x^{2}}t^{3\alpha }}{\Gamma (3\alpha +1)},\\&u_{4}=\frac{e^{x^{2}}t^{4\alpha }}{\Gamma (4\alpha +1)},\\&\ldots \ldots \\&u_{n}=\frac{e^{x^{2}}t^{n\alpha }}{\Gamma (n\alpha +1)}. \end{aligned}$$

The solution of Eq. (25) is given as

28 $$\begin{aligned} u(x,t)&=e^{x^{2}}\left( 1+\frac{t^{\alpha }}{\Gamma (\alpha +1)}+\frac{t^{2\alpha }}{\Gamma (2\alpha +1)} +\frac{t^{3\alpha }}{\Gamma (3\alpha +1)}+\cdots +\frac{t^{n\alpha }}{\Gamma (n\alpha +1)}\right) \nonumber \\&=e^{x^{2}}E_{\alpha }(t^{\alpha }). \end{aligned}$$

The series (28) is approximate to the form $u(x,t)=e^{x^{2}+t}$ for $\alpha =1$, which is the exact solution of Eq. (25) for $\alpha =1$. The result is complete agreement with HPTM (Kumar 2013).

### *Example 3*

Consider the following time-fractional Cauchy reaction–diffusion equation (Kumar 2013) as follows

29 $$\left\{ \begin{array}{lll} &{}u_{t}^{\alpha }(x,t)=u_{xx}u(x,t)-(4x^{2}-2t+2)u(x,t),\\ &{}0<\alpha \le 1,\\ &{}u(x,0)=e^{x^{2}}. \end{array} \right.$$

Applying Sumudu transform on both sides of Eq. (29) and using the differential property of Sumudu transform, we obtain

30 $$\begin{aligned} S[u(x,t)]=u(x,0)+v^{\alpha }S\left[ u_{xx}(x,t)-(4x^{2}-2t+2)u(x,t)\right] . \end{aligned}$$

Using the inverse Sumudu transform on both sides of Eq. (30), we can get

31 $$\begin{aligned} u(x,t)=e^{x^{2}}+S^{-1}\left[ v^{\alpha }S[u_{xx}(x,t)-(4x^{2}-2t+2)u(x,t)]\right] . \end{aligned}$$

According to the NSTIM, we have

$$\left\{ \begin{array}{ll} &{}u_{0}=e^{x^{2}},\\ &{}K[u(x,t)]=S^{-1}\left[ v^{\alpha }S[u_{xx}(x,t)-(4x^{2}-2t+2)u(x,t)]\right] . \end{array} \right.$$

By iteration, the following results are obtained

$$\begin{aligned}&u_{0}=e^{x^{2}},\\&u_{1}=2e^{x^{2}}\frac{t^{\alpha +1}}{\Gamma (\alpha +2)},\\&u_{2}=4e^{x^{2}}\frac{\Gamma (\alpha +3)t^{2\alpha +2}}{\Gamma (\alpha +2)\Gamma (2\alpha +3)},\\&u_{3}=8e^{x^{2}}\frac{\Gamma (\alpha +3)\Gamma (2\alpha +4)t^{3\alpha +3}}{\Gamma (\alpha +2) \Gamma (2\alpha +3)\Gamma (3\alpha +4)},\\&u_{4}=16e^{x^{2}}\frac{\Gamma (\alpha +3)\Gamma (2\alpha +4)\Gamma (3\alpha +5) t^{4\alpha +4}}{\Gamma (\alpha +2)\Gamma (2\alpha +3)\Gamma (3\alpha +4)\Gamma (4\alpha +5)},\\&\ldots \end{aligned}$$

Thus, the solution of Eq. (29) can be written in the following form

32 $$\begin{aligned} u&=u_{0}+u_{1}+u_{2}+u_{3}+u_{4}+\cdots \nonumber \\&=e^{x^{2}}+2e^{x^{2}}\frac{t^{\alpha +1}}{\Gamma (\alpha +2)}+4e^{x^{2}}\frac{\Gamma (\alpha +3)t^{2\alpha +2}}{\Gamma (\alpha +2)\Gamma (2\alpha +3)}\nonumber \\&\quad +\,8e^{x^{2}}\frac{\Gamma (\alpha +3)\Gamma (2\alpha +4)t^{3\alpha +3}}{\Gamma (\alpha +2) \Gamma (2\alpha +3)\Gamma (3\alpha +4)} \nonumber \\&\quad +\,16e^{x^{2}}\frac{\Gamma (\alpha +3)\Gamma (2\alpha +4)\Gamma (3\alpha +5)t^{4\alpha +4}}{\Gamma (\alpha +2)\Gamma (2\alpha +3)\Gamma (3\alpha +4)\Gamma (4\alpha +5)}+\cdots \end{aligned}$$

The exact solution of Eq. (29) is $u(x,t)=e^{x^{2}+t^{2}}$ for $\alpha =1$.

### *Remark 1*

 The above are three examples of the linear fractional Cauchy reaction–diffusion equation. Figures 1, 2, 3, 4, 5, 6, 7, 8, 9, 10, 11 and 12 respectively show the approximate solutions of the linear fractional Cauchy reaction–diffusion equations at different values for $\alpha =0.6, 0.8, 1$ and the exact solutions for $\alpha =1$. It is very easy to find that the solution continuously depend on the values of time-fractional derivative.

**Fig. 1:**
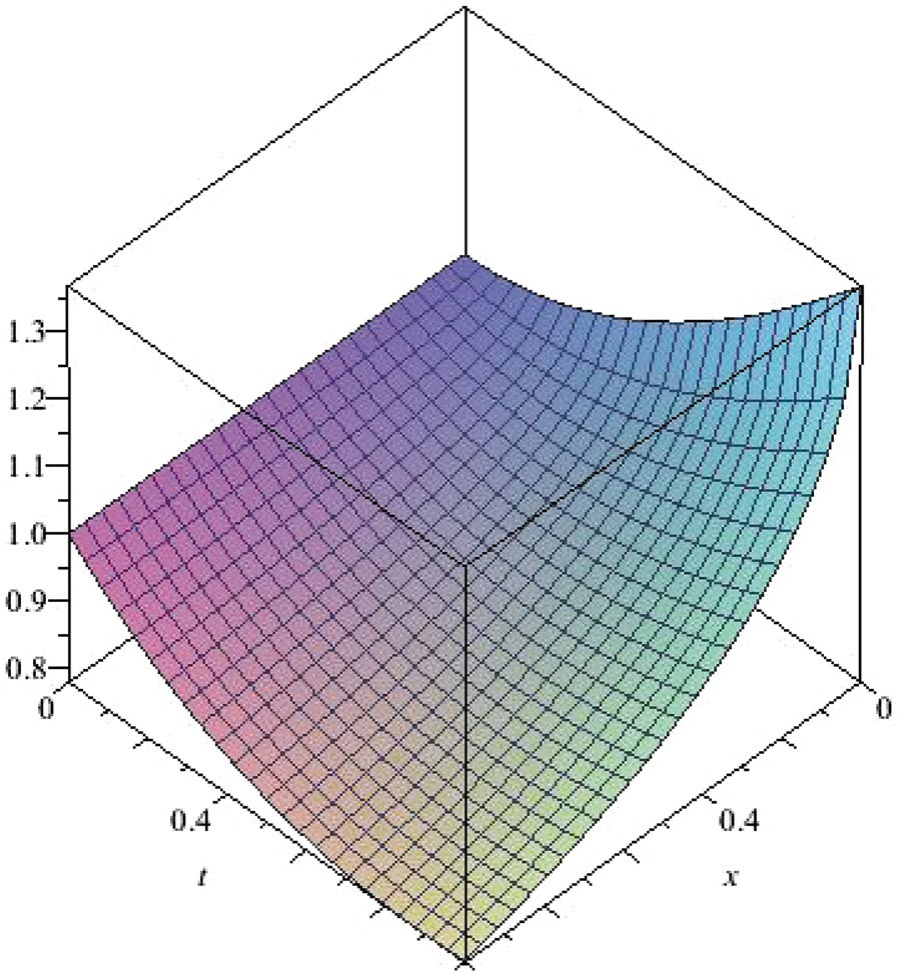
The 10th-order approximate solution of Eq. (20) for $\alpha =0.6$

**Fig. 2:**
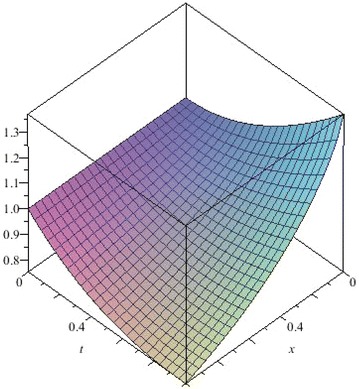
The 10th-order approximate solution of Eq. (20) for $\alpha =0.8$

**Fig. 3:**
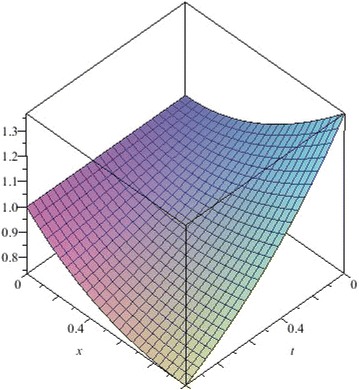
The 10th-order approximate solution of Eq. (20) for $\alpha =1$

**Fig. 4:**
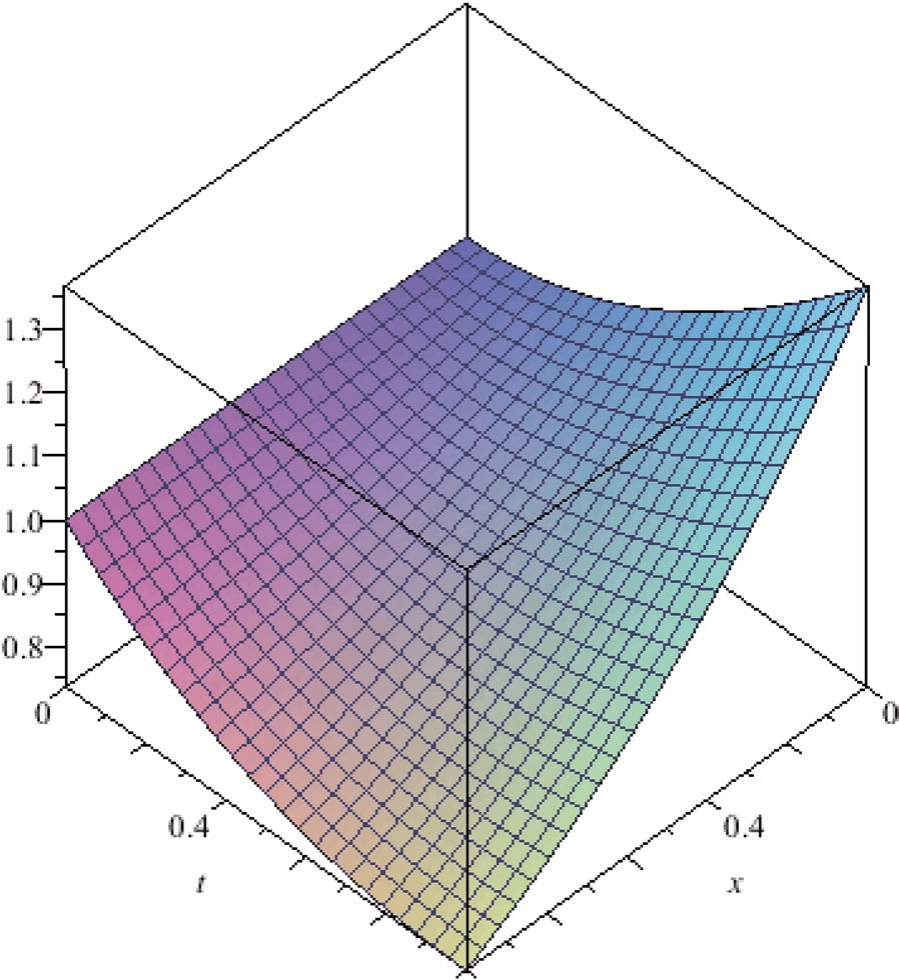
The exact solution of Eq. (20) for $\alpha =1$

**Fig. 5:**
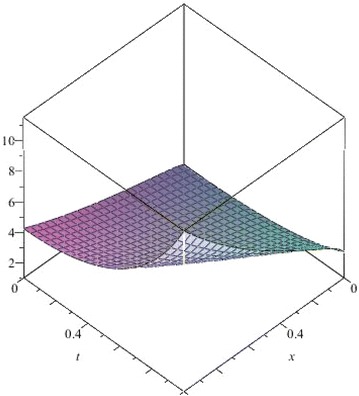
The 10th-order approximate solution of Eq. (25) for $\alpha =0.6$

**Fig. 6:**
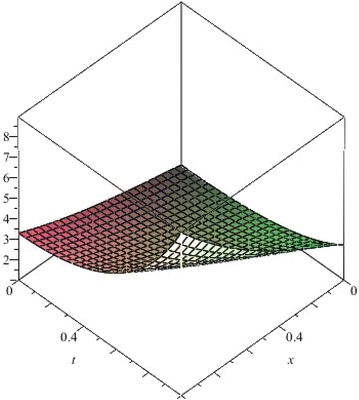
The 10th-order approximate solution of Eq. (25) for $\alpha =0.8$

**Fig. 7:**
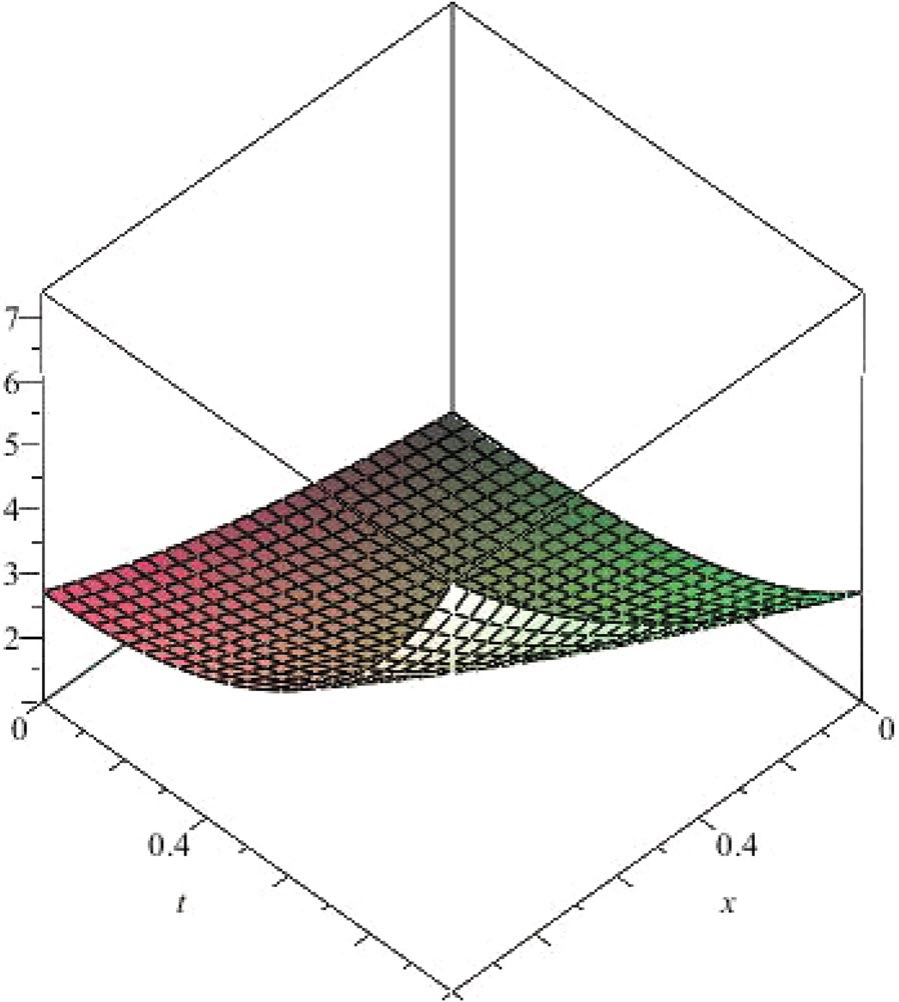
The 10th-order approximate solution of Eq. (25) for $\alpha =1$

**Fig. 8:**
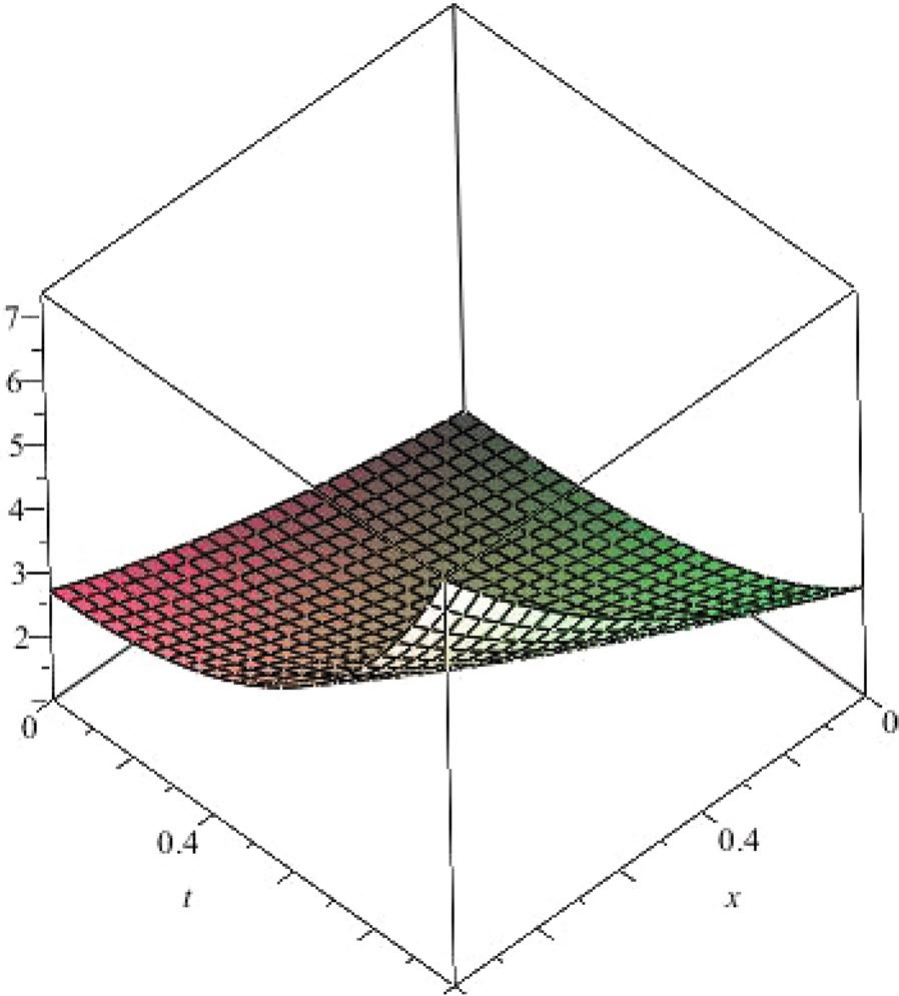
The exact solution of Eq. (25) for $\alpha =1$

**Fig. 9:**
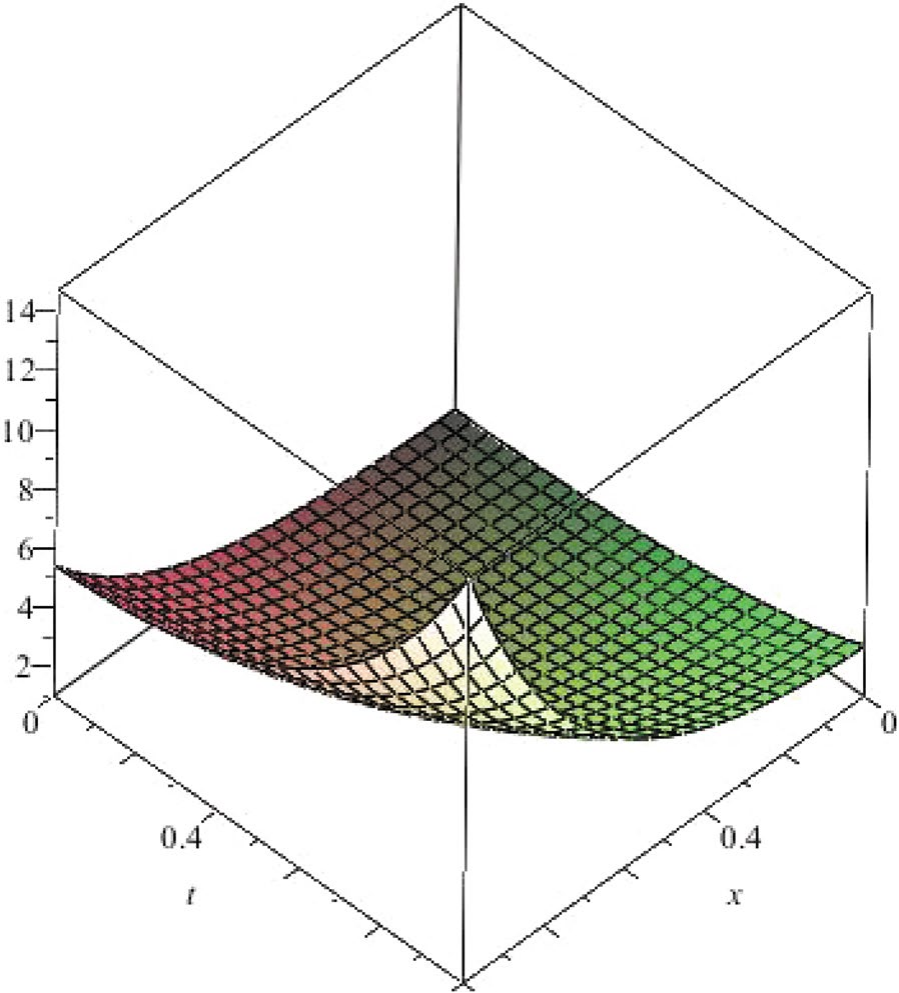
The 5th-order approximate solution of Eq. (29) for $\alpha =0.6$

**Fig. 10:**
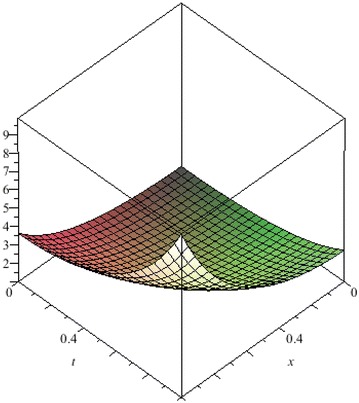
The 5th-order approximate solution of Eq. (29) for $\alpha =0.8$

**Fig. 11:**
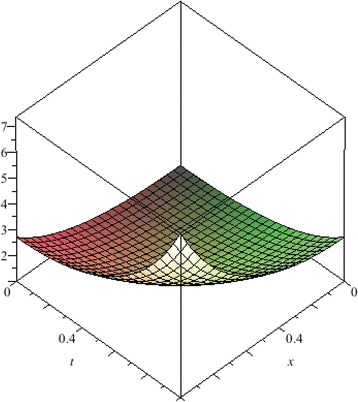
The 5th-order approximate solution of Eq. (29) for $\alpha =1$

**Fig. 12:**
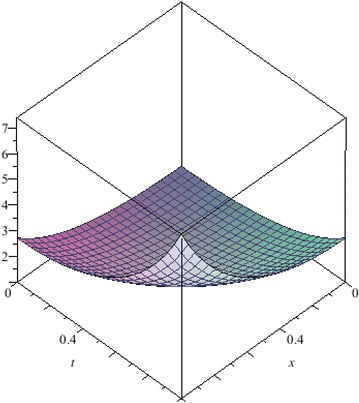
The exact solution of Eq. (29) for $\alpha =1$

### *Remark 2*

**Fig. 13:**
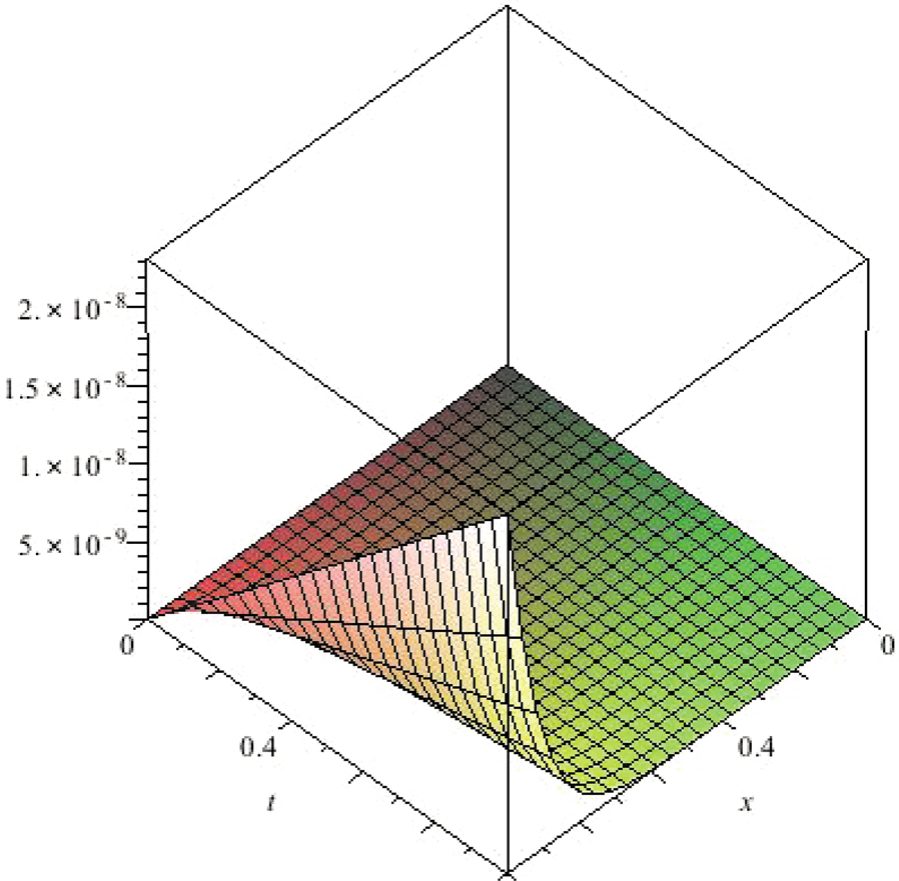
The absolute error $|u_{exa}-u_{10app}|$ of Eq. (20) for $\alpha =1$

**Fig. 14:**
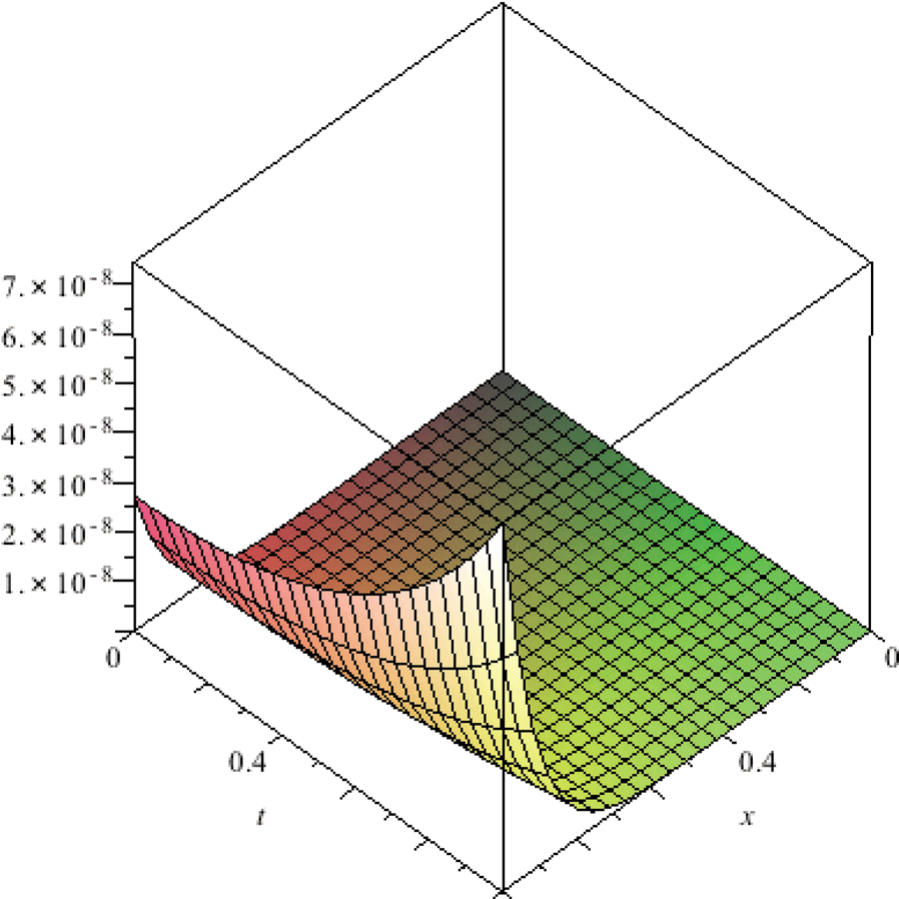
The absolute error $|u_{exa}-u_{10app}|$ of Eq. (25) for $\alpha =1$

**Fig. 15:**
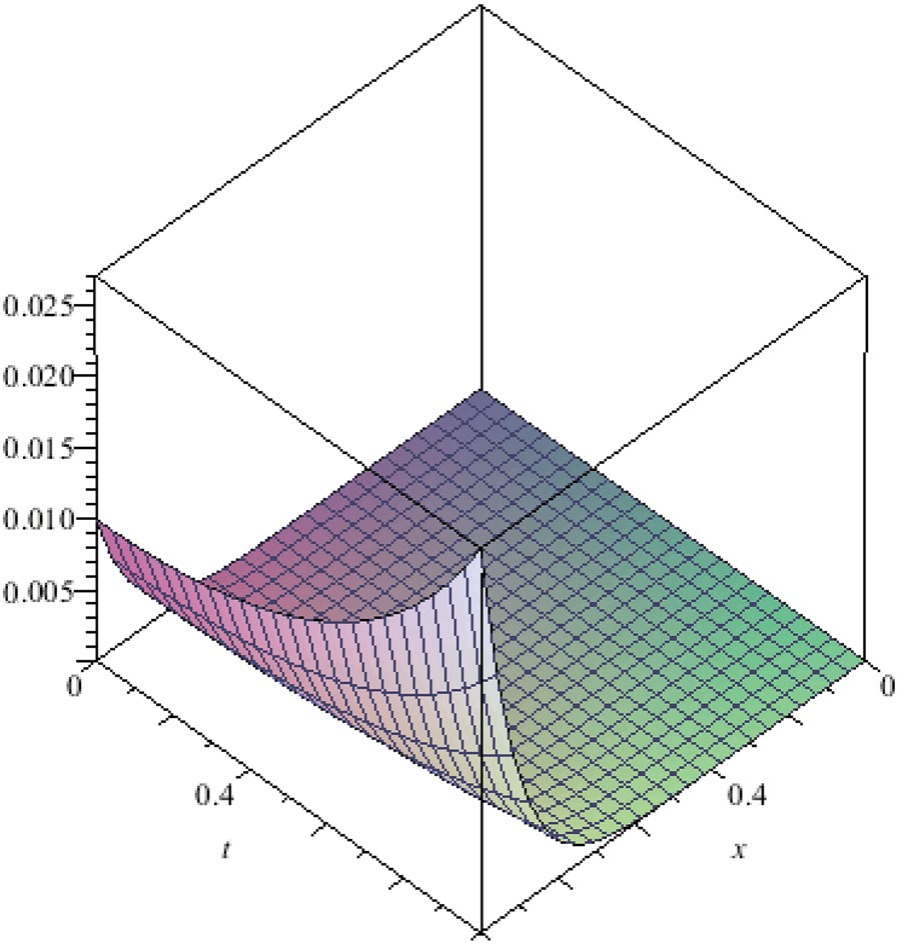
The absolute error $|u_{exa}-u_{5app}|$ of Eq. (29) for $\alpha =1$

Figures 13, 14 and 15 show the absolute error between approximate solutions and exact solutions for $\alpha =1$. In Tables 1, 2 and  3, we compute the approximate solutions and the exact solutions at different points for $\alpha =1$. By comparison, we find that it is evident the accuracy and efficiency of this method can be dramatically enhanced by computing further terms. In this paper, we only use several terms. If we use more terms, the accuracy of the approximate solution will be greatly improved. Therefore, the proposed method is accurate and efficient for linear differential equation.

**Table 1:** Comparison between the 10th-order approximate solution of Eq. (20) and the exact solution for $\alpha =1$

*x*	*t*	$u_{exa}$	$u_{NSTIM}$	$|u_{exa}-u_{10app}|$
$$\alpha =1$$				
0.2	0.3	0.9668943972	0.9668943972	$0.0\times 10^{-10}$
0.4	0.3	0.9666473343	0.9666473342	$1.0\times 10^{-10}$
0.5	0.6	0.8809364777	0.8809364778	$1.0\times 10^{-10}$
0.7	0.8	0.8111155787	0.8111155801	$1.4\times 10^{-9}$

**Table 2:** Comparison between the 10th-order approximate solution of Eq. (25) and the exact solution for $\alpha =1$

*x*	*t*	$u_{exa}$	$u_{NSTIM}$	$|u_{exa}-u_{10app}|$
$$\alpha =1$$				
0.3	0.4	1.632316221	1.632316221	$0.0\times 10^{-9}$
0.5	0.6	2.339646852	2.339646853	$1.0\times 10^{-9}$
0.6	0.7	2.886370989	2.886370989	$0.0\times 10^{-9}$
0.7	0.8	3.632786555	3.632786553	$2.0\times 10^{-9}$

**Table 3:** Comparison between the 5th-order approximate solution of Eq. (29) and the exact solution for $\alpha =1$

*x*	*t*	$u_{exa}$	$u_{NSTIM}$	$|u_{exa}-u_{5app}|$
$$\alpha =1$$				
0.2	0.3	1.138828383	1.138828331	0.000000052
0.4	0.5	1.506817785	1.506807822	0.000009963
0.3	0.6	1.568312186	1.568253566	0.000058620
0.7	0.8	3.095656500	3.094024665	0.001631835

### *Example 4*

In this example, we consider the nonlinear fractional Cauchy reaction–diffusion equation (Momani and Yildirim 2010) as follows

33 $$\left\{ \begin{array}{lll} &{}u_{t}^{\alpha }u=u_{xx}-u_{x}+uu_{xx}-u^{2}+u,\\ &{}0<\alpha \le 1,\\ &{}u(x,0)=e^{x}. \end{array} \right.$$

Operating the Sumudu transform on both sides of Eq. (33) and applying the property of Sumudu transform for fractional derivative, we get

34 $$\begin{aligned} S[u]=u(x,0)+v^{\alpha }S[u_{xx}-u_{x}+uu_{xx}-u^{2}+u]. \end{aligned}$$

Using the inverse Sumudu transform technique on the both sides of Eq. (34), we have

35 $$\begin{aligned} u(x,t)=e^{x}+S^{-1}[v^{\alpha }S[u_{xx}-u_{x}+u]]+S^{-1}[v^{\alpha }S[uu_{xx}-u^{2}]]. \end{aligned}$$

According to the NSTIM, we get

$$\left\{ \begin{array}{lll} &{}u_{0}=e^{x},\\ &{}K[u(x,t)]=S^{-1}[v^{\alpha }S[u_{xx}-u_{x}+u]],\\ &{}N[u(x,t)]=S^{-1}[v^{\alpha }S[uu_{xx}-u^{2}]]. \end{array} \right.$$

By iteration, the following result is obtained

$$\begin{aligned}&u_{0}=e^{x},\\&u_{1}=\frac{e^{x}t^{\alpha }}{\Gamma (\alpha +1)},\\&u_{2}=\frac{e^{x}t^{2\alpha }}{\Gamma (2\alpha +1)},\\&u_{3}=\frac{e^{x}t^{3\alpha }}{\Gamma (3\alpha +1)},\\&u_{4}=\frac{e^{x}t^{4\alpha }}{\Gamma (4\alpha +1)},\\&\ldots \ldots \\&u_{n}=\frac{e^{x}t^{n\alpha }}{\Gamma (n\alpha +1)}. \end{aligned}$$

Therefore, the solution of the Eq. (33) is given as

36 $$\begin{aligned} u(x,t)&=e^{x}\left(1+\frac{t^{\alpha }}{\Gamma (\alpha +1)}+\frac{t^{2\alpha }}{\Gamma (2\alpha +1)} +\frac{t^{3\alpha }}{\Gamma (3\alpha +1)}+\cdots +\frac{t^{n\alpha }}{\Gamma (n\alpha +1)}\right) \\& =e^{x}E_{\alpha }(t^{\alpha })\end{aligned}$$

The result is complete agreement with HPM (Momani and Yildirim 2010). when $\alpha =1$, (36) can be expressed into the following form as

37 $$\begin{aligned} u(x,t)=e^{x}E(t)=\sum _{k=0}^{\infty }\frac{t^{k}}{\Gamma (1+k)}=e^{x}\sum _{k=0}^{\infty }\frac{t^{k}}{k!}=e^{x+t}. \end{aligned}$$

### *Remark 3*

The Eq. (37) is the exact solution of Eq. (33) for $\alpha =1$.

### *Remark 4*

**Fig. 16:**
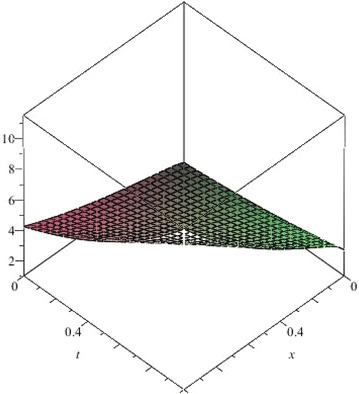
The 10th-order approximate solution of Eq. (33) for $\alpha =0.6$

**Fig. 17:**
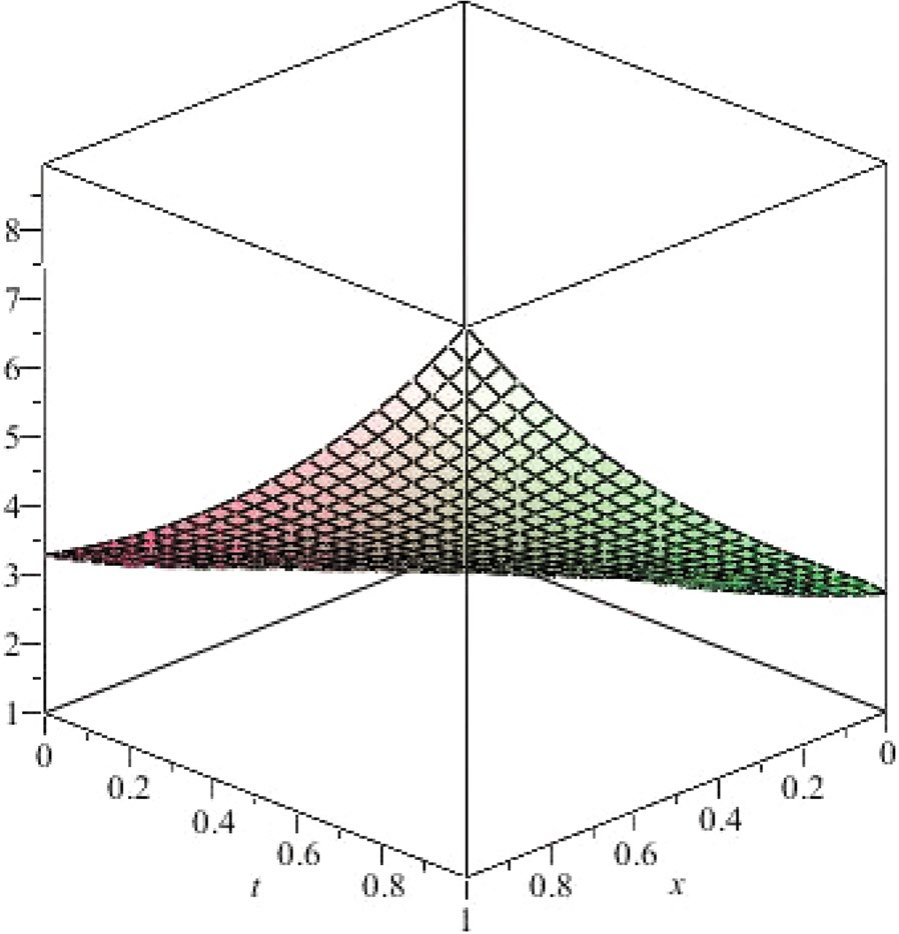
The 10th-order approximate solution of Eq. (33) for $\alpha =0.8$

**Fig. 18:**
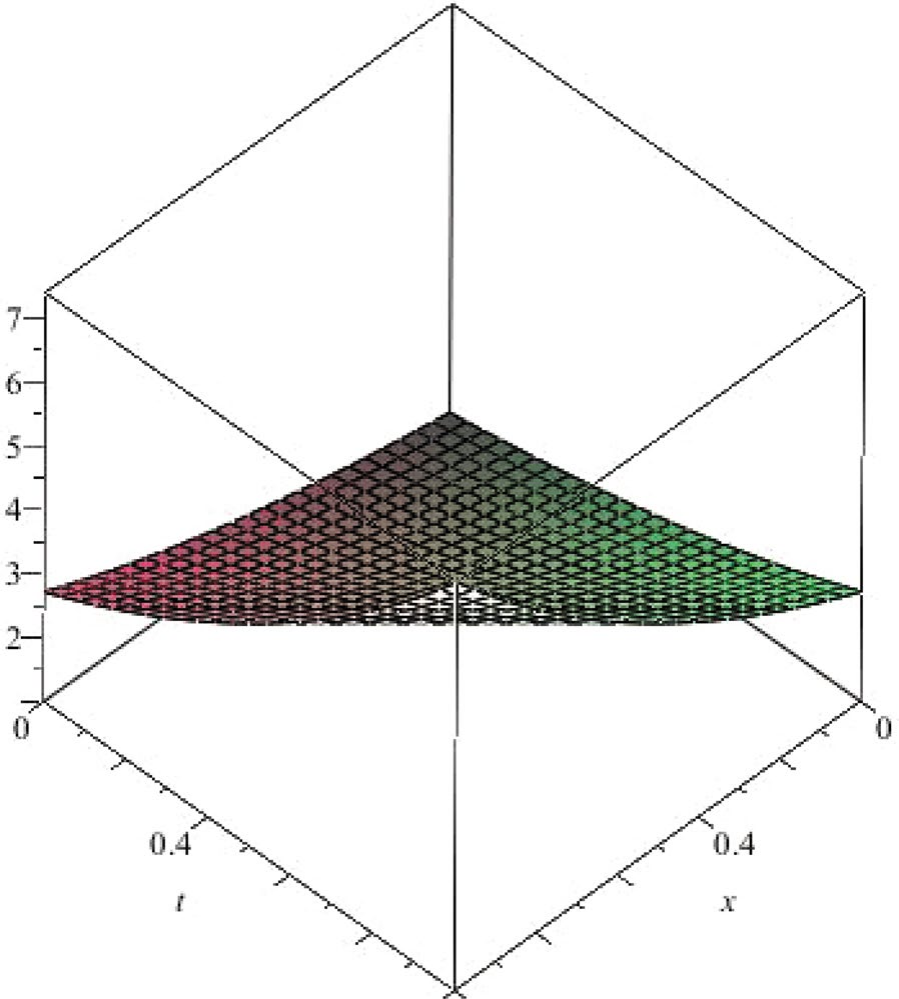
The 10th-order approximate solution of Eq. (33) for $\alpha =1$

**Fig. 19:**
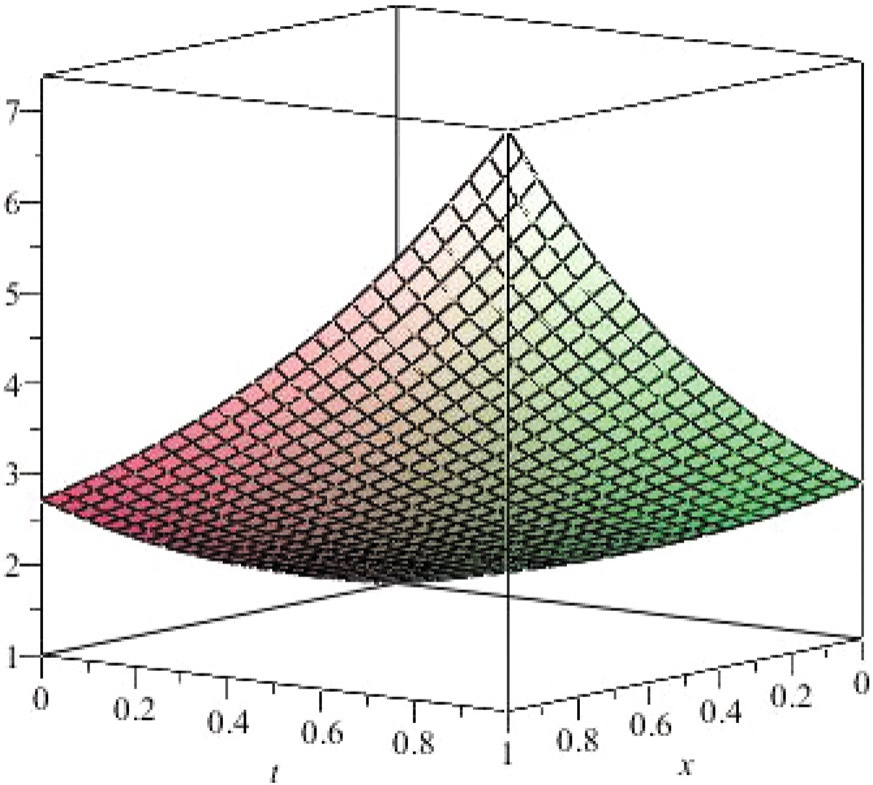
The exact solution of Eq. (33) for $\alpha =1$

**Fig. 20:**
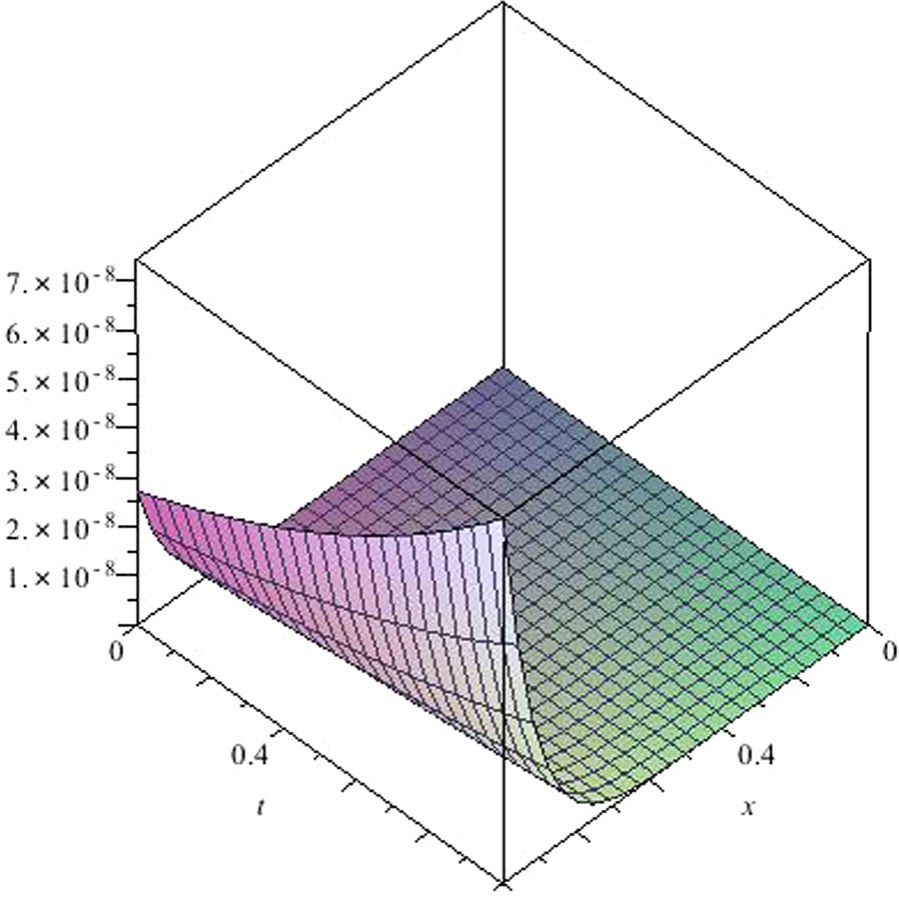
The absolute error $|u_{exa}-u_{10app}|$ of Eq. (33) for $\alpha =1$

In this example, we apply the NSTIM to solve the nonlinear Cauchy reaction–diffusion equation. In Table 4, we compute the different values between the 10th-order approximate solution and the exact solution of Eq. (33) for $\alpha =1$. Figs. 16, 17, 18 and 19 show 10th-order approximate solutions for $\alpha =0.6,\,\alpha =0.8,\,\alpha =1$, and the exact solution of Eq. (33). Figure 20 show the absolute error between approximate solution and exact solution for $\alpha =1$. By comparing Table 4 with Figs. 16, 17, 18 and 19, we can find the NSTIM is very accurate and efficient to solve the nonlinear Cauchy reaction-equation. The accuracy of this method depends on the number of terms. So, the NSTIM is a very efficient method to solve the nonlinear fractional differential equation.

**Table 4:** Comparison between the 10th-order approximate solution of Eq. (33) and the exact solution for $\alpha =1$

*x*	*t*	$u_{exa}$	$u_{NSTIM}$	$|u_{exa}-u_{10app}|$
$$\alpha =1$$				
0.2	0.2	1.491824698	1.491824698	$0.0\times 10^{-9}$
0.4	0.3	2.013752707	2.013752707	$0.0\times 10^{-9}$
0.6	0.4	2.718281828	2.718281129	$1.0\times 10^{-9}$
0.7	0.8	4.481689070	4.481689066	$4.0\times 10^{-9}$

## Conclusion

In this paper, the new Sumudu transform iterative method has been successfully applied for finding the approximate solution for the time-fractional Cauchy reaction–diffusion equation. The advantage of the new Sumudu transform iterative method (NSTIM) is to combine new iterative method (NIM) and Sumudu transform for obtaining exact and approximate analytical solutions for the time-fractional Cauchy reaction–diffusion equations.The numerical results show that the Sumudu transform iterative method is highly efficient and accurate with less calculation than existing methods.
